# Validation of a smartphone-based device to measure concentration, motility, and morphology in swine ejaculates

**DOI:** 10.1093/tas/txac119

**Published:** 2022-08-28

**Authors:** Aridany Suárez-Trujillo, Hemanth Kandula, Jasmine Kumar, Anjali Devi, Larissa Shirley, Prudhvi Thirumalaraju, Manoj Kumar Kanakasabapathy, Hadi Shafiee, Liane Hart

**Affiliations:** Department of Animal Science, Purdue University, West Lafayette, IN 47909, USA; Department of Animal Science, Berry College, Mount Berry, GA 30149, USA; Division of Engineering in Medicine, Department of Medicine, Brigham and Women’s Hospital, Harvard Medical School, Boston, MA 02139, USA; Division of Engineering in Medicine, Department of Medicine, Brigham and Women’s Hospital, Harvard Medical School, Boston, MA 02139, USA; Division of Engineering in Medicine, Department of Medicine, Brigham and Women’s Hospital, Harvard Medical School, Boston, MA 02139, USA; Department of Animal Science, Purdue University, West Lafayette, IN 47909, USA; Division of Engineering in Medicine, Department of Medicine, Brigham and Women’s Hospital, Harvard Medical School, Boston, MA 02139, USA; Division of Engineering in Medicine, Department of Medicine, Brigham and Women’s Hospital, Harvard Medical School, Boston, MA 02139, USA; Division of Engineering in Medicine, Department of Medicine, Brigham and Women’s Hospital, Harvard Medical School, Boston, MA 02139, USA; Verility, Inc., Maxwell, IN 46154, USA

**Keywords:** smartphone, based semen analysis, swine

## Abstract

Assessment of swine semen quality is important as it is used as an estimate of the fertility of an ejaculate. There are many methods to measure sperm morphology, concentration, and motility, however, some methods require expensive instrumentation or are not easy to use on-farm. A portable, low-cost, automated device could provide the potential to assess semen quality in field conditions. The objective of this study was to validate the use of Fertile-Eyez (FE), a smartphone-based device, to measure sperm concentration, total motility, and morphology in boar ejaculates. Semen from six sexually mature boars were collected and mixed to create a total of 18 unique semen samples for system evaluations. Each sample was then diluted to 1:4, 1:8, 1:10, and 1:16 (for concentration only) with Androhep Plus semen extender (*n* = 82 total). Sperm concentration was evaluated using FE and compared to results measured using a Nucleocounter and computer assisted sperm analysis (CASA: Ceros II, Hamilton Thorne). Sperm motility was evaluated using FE and CASA. Sperm morphological assessments were evaluated by a single technician manually counting abnormalities and compared to FE deep-learning technology. Data were analyzed using both descriptive statistics (mean, standard deviation, intra-assay coefficient of variance, and residual standard deviation [RSD]) and statistical tests (correlation analysis between devices and Bland-Altman methods). Concentration analysis was strongly correlated (*n* = 18; *r* > 0.967; *P* < 0.0001) among all devices and dilutions. Analysis of motility showed moderate correlation and was significant when all dilutions are analyzed together (*n* = 54; *r* = 0.558; *P* < 0.001). The regression analysis for motility also showed the RSD as 3.95% between FE and CASA indicating a tight fit between devices. This RSD indicates that FE can find boars with unacceptable motility (boars for example with less than 70%) which impact fertility and litter size. The Bland-Altman analysis showed that FE-estimated morphological assessment and the conventionally estimated morphological score were similar, with a mean difference of ~1% (%95 Limits of Agreement: −6.2 to 8.1; *n* = 17). The results of this experiment demonstrate that FE, a portable and automated smartphone-based device, is capable of assessing concentration, motility, and morphology of boar semen samples.

## INTRODUCTION

Evaluation of boar semen for measures of semen quality is an important component to success when using artificial insemination. Immediately following ejaculation, semen is evaluated for volume, concentration, motility, and morphological abnormalities of the sperm cells. The use of poor-quality semen, with low concentration, motility, or high number of morphological abnormalities, is correlated with low reproductive success after insemination (Flowers, 1997). There are multiple methods to asses concentration of sperm cells within an ejaculate, most commonly, direct cell counting using a hemocytometer (Jasko, 1992) or spectrophotometry (Camus et al., 2011). These two methods are relatively inexpensive, however, hemocytometer hand counting of sperm cells can be time-consuming limiting its practicality for use on every ejaculate in a commercial setting (Dini et al., 2019). Additional methods include nuclear staining to differentiate sperm cells from other particles Nucleocounter (NC) SP-100 (Morrell et al., 2010), cell sorting using flow cytometry with fluorescent cell labeling (Hansen et al., 2006), or computer-assisted sperm analysis (CASA; Zinaman et al., 1996, Amann and Waberski, 2014). These methods can be highly accurate; however, they require either expensive instruments or may not be adaptable for farm-based use. A portable, low-cost, automated device that could be used in field settings or for smaller operations could be useful if accurate and repeatable.

Smartphone-based devices for semen evaluation have been previously tested in humans, stallions, and dogs (Kanakasabapathy et al., 2017, Thirumalaraju et al., 2018, Buss et al., 2019, Dini et al., 2019, Thirumalaraju et al., 2019, Bulkeley et al., 2021, Kanakasabapathy et al., 2021). Those smartphone-based devices, similar to CASA systems, use images captured from semen samples loaded in a chamber slide to evaluate sperm cell concentration, motility, and morphology (Mortimer et al., 2015).

The aim of this study was to evaluate the accuracy and repeatability of measures of sperm concentration, motility, and morphology in boar ejaculates using Fertile-Eyez (FE), a smartphone-based semen evaluation device. To achieve this aim, the concentration of sperm cells in ejaculates and motility were compared to two devices generally accepted as highly repeatable and accurate in the swine industry, the NC SP-100 and CASA (Ceros II, Hamilton Thorne, USA). Morphology estimations were compared to conventional manual assessments performed using phase-contrast microscopy.

## MATERIALS AND METHODS

### Animals

Six sexually mature boars (20 months old) with known semen quality above 75% total motility and 85% morphologically normal sperm cells were used for this study. Boars were collected under a protocol reviewed and approved by Purdue University Institutional Animal Care and Use Committee (#2012002099). Boars were housed in individual stalls and fed a maintenance diet once per day.

### Semen Collection and Dilutions

Semen was collected using the double-gloved-hand method to minimize bacterial contamination of the ejaculates. Ejaculates from six boars were collected and mixed in pairs to create a total of 18 unique semen samples. The main objective of mixing semen samples to create 18 unique samples was to increase the sample size for this study. Mixed ejaculates were then diluted with Androhep Plus (Minitube, USA) semen extender to dilutions of 1:4, 1:8, 1:10, and 1:16. Extended semen samples were placed into a cooler at 17 °C overnight until analysis. All dilutions were used for determination of concentration, but only concentration 1:4, 1:8, and 1:10 were used for determination of motility. Dilution 1:16 was not used for motility determination due to low number of sperm cells per field to have a representative number sample for this determination. CASA system settings would need to have been adjusted to accurately measure at this low level of sperm.

### Determination of Sperm Concentration and Total Motility

#### NC.

Semen samples were diluted with Reagent S100 following manufacturer recommendations based on the appropriate dilution factor, vortexed for 10 s and loaded into an SP-100 cassette. The cassette was then inserted into the NC SP-100 machine and evaluated for the total number of sperm cells. Each sample was evaluated in triplicate by loading three individual cartridges from the same semen/Reagent S100 mix.

#### FE.

FE device is a smartphone-based device developed by Kanakasabapathy et al. (2017) and Kanakasabapathy et al. (2021). Briefly, an optical hardware smartphone attachment, composed of a pair of lenses, a small battery, an LED light, and a 3-D printed support base, was used for sperm cells imaging. The recorded videos of fresh semen samples and images of smeared stained sperm cells were used for measuring sperm concentration, motility, and morphology using a deep learning-based framework. A 2-mL aliquot of each diluted ejaculate was transferred to a clean polypropylene tube and incubated at 37 °C for 20 min. After warming, the sample was mixed by hand and 3 µL loaded in a pre-warmed 2-chamber slide (Leja, IMV, USA). The chamber slide was then inserted into the support base of the FE device for analysis of concentration and total motility. The smartphone application records 1s duration videos (30 fps) and processes each frame to obtain sperm concentration and motility.

#### CASA.

The same warmed 2 mL sample of semen was used to evaluate concentration and motility on the CASA system (Ceros II, Hamilton Thorne, USA). A 3 µL aliquot was loaded into a 4-chamber slide (Leja, IMV, USA) and placed on a warmed (37 °C) microscope stage of an AxioLAB A1 Zeiss microscope equipped with a 20× FINH objective. Within each chamber, six fields were analyzed and the average concentration and motility reported. Each sample was analyzed in triplicate using three slide chambers.

### Morphology Assessment

A subsample (1 mL) of each semen mixture was preserved with 100 µL of 10% formalin for evaluation of sperm cell morphology. Using phase-contrast, bright-field microscopy (40×), 200 randomly selected sperm cells were categorized as morphologically normal or containing proximal or distal cytoplasmic droplets, distal midpiece reflex, abnormal heads, or tails. Each of the 18 semen samples was manually counted a single time by a single technician. The preserved samples were then mixed with eosin stain, smeared on a cleaned glass slide, covered with a glass coverslip, and sealed with clear nail polish. Stained slides were shipped to Dr. Shafiee’s laboratory at Brigham and Women’s Hospital where the FE deep learning technology was used to evaluate sperm cell morphological abnormalities. The smeared microscope slide was inserted into the device, similarly to the Leja slide for motility analysis, and evaluated for morphological abnormalities using a deep learning algorithm.

Python 3.6 using PyTorch (v1.5.0) was used to implement the deep learning algorithm used in this study (MDnets) (Kanakasabapathy et al., 2021) and public libraries such as OS, time, csv, sklearn, math, copy, Itertools, random, and NumPy were used. The network was built on a computer running Ubuntu 18.04 Linux. The network training was GPU-bound, and the training was performed using 3 GeForce GTX 1080Ti GPUs (Nvidia). The MDnet framework consists of a base network architecture with a final flattened layer linked to a classifier block and an adversarial block. MD-nets are trained by limiting the classification loss created by the classification block using the source data while maximizing the discriminator loss, which increases domain confusion (Kanakasabapathy et al., 2021). One of the 18 samples was randomly selected to be used as a control sample for device calibration, leaving 17 samples evaluated by both the technician and FE technologies.

Individual sperm images annotated through manual assessment by expert-technical staff was used to evaluate the trained algorithm at the single-cell level. We utilized images collected using a benchtop microscope for this section of the analysis similar to a previous study by Kanakasabapathy et al. (2021). The algorithm was evaluated using 270 individual sperm cell images and through a receiver operating characteristic (ROC) analysis, an area under the curve (AUC) of 0.994 (*P* < 0.001) was obtained, which indicated that the algorithm excelled at differentiating between sperm cells based on their morphology (normal vs. abnormal).

### Statistical Analysis

All analyses were performed using SAS v9.4 (SAS Institute, Cary, NC), Prism v9.2 (Graphpad, CA), and MedCalc v20.009 (MedCalc Software, Belgium). Statistical analyses were performed in agreement with previous research testing similar devices (Dini et al., 2019). The mean and standard deviation (SD) were calculated from the three replicates for each diluted semen sample. These factors were used to calculate the coefficient of variation (CV) as an evaluation of repeatability. Accuracy assessments were performed using Pearson correlation coefficients in PROC CORR and linear regression analysis was performed with PROC REG, both in SAS. Band-Altman plots were created by comparing the difference in response (concentration, motility, or morphology) between two methods for each sample and at each dilution (concentration and motility only), to compare similarities between the two approaches (Bland and Altman, 1986). Statistical significance was established as *P* ≤ 0.05 and *P*-values > 0.05 and ≤0.10 were considered a tendency. Coefficients of correlation greater than 0.40 were considered moderately correlated, and coefficients greater than 0.70 were considered as strongly correlated (Ratner, 2009, Mukaka, 2012, Schober et al., 2018).

## RESULTS AND DISCUSSION

### Assessment of Sperm Concentration

The results for evaluation of concentration for the three devices at all four dilutions are shown in Table 1. The descriptive statistics SD and intra-assay CV provide information about the repeatability of the instruments. The NC had the lowest SD (range 0.54–1.72) and CV (range 1.98%–3.4%) for concentration at all dilutions. The CASA system and FE had similar SD (range for CASA 3.0–9.39 and FE 2.49–8.58) and CV (range for CASA 7.28–15.36 and FE 11.15–16.97). Analysis of canine sperm concentration with an iPad-based device found similar to slightly higher CV (22.97%) when the repeatability of the concentration was assessed (Bulkeley et al., 2021).

**Table 1.** Mean concentration (10^6^ cells/mL) and coefficient of variation (CV) of serial-diluted swine semen samples measured with Nucleocounter, Fertile-Eyez, and CASA

	Nucleocounter			Fertile-Eyez			CASA		
Dilution	Mean	SD	CV	Mean	SD	CV	Mean	SD	CV
1:4	87.88	1.72	1.98	76.22	8.58	11.15	84.80	6.07	7.28
1:8	37.89	0.78	2.03	35.96	6.05	15.04	36.26	3.62	10.26
1:10	26.65	0.87	3.40	21.39	3.56	16.97	30.56	9.39	13.80
1:16	19.26	0.54	2.81	16.39	2.49	15.31	18.83	3.00	15.36

**Figure 1.**
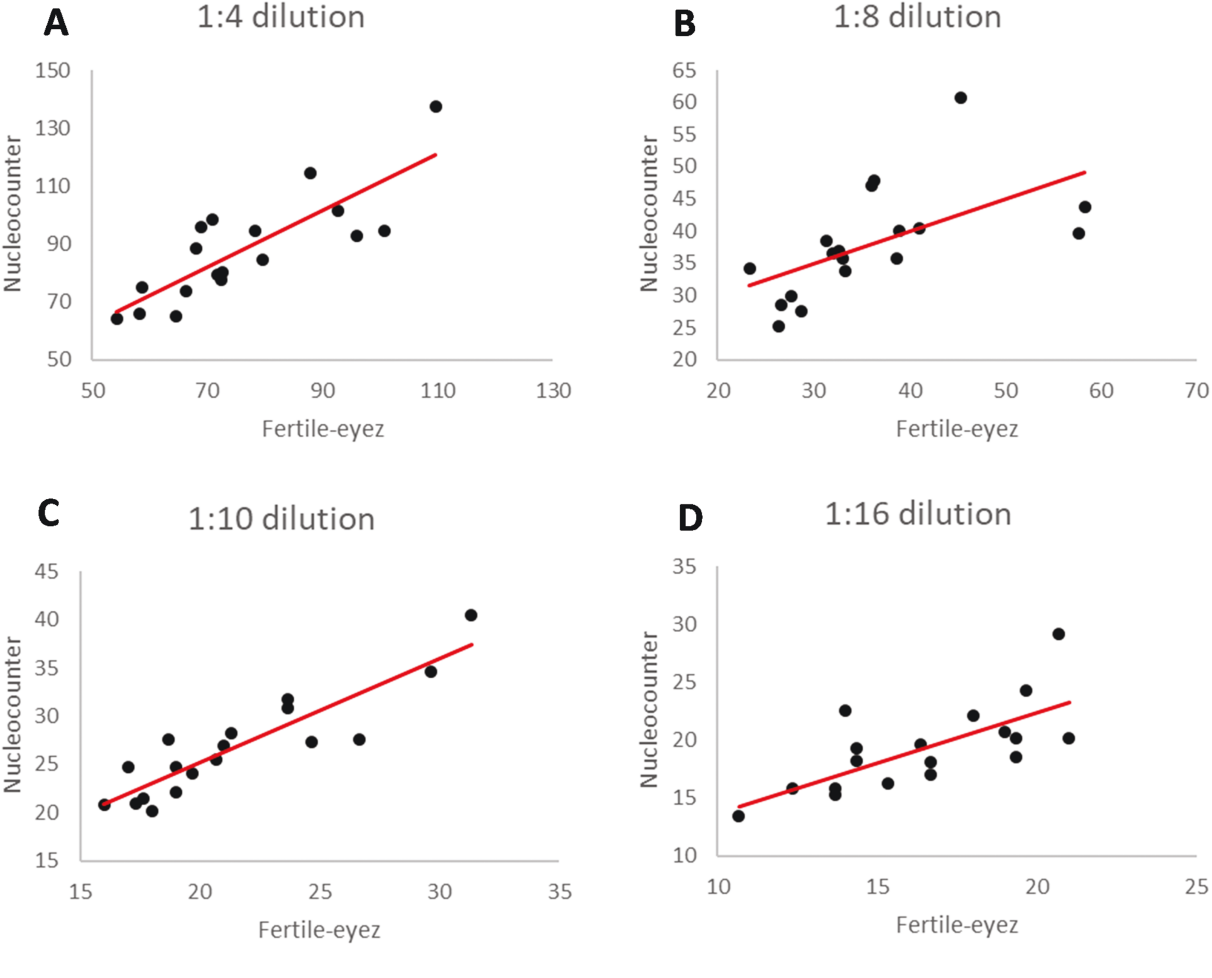
Linear regression between sperm concentration values measured with Fertile-Eyez, compared to Nucleocounter at 1:4 (A), 1:8 (B), 1:10 (C), and 1:16 (D) dilution.

**Figure 2.**
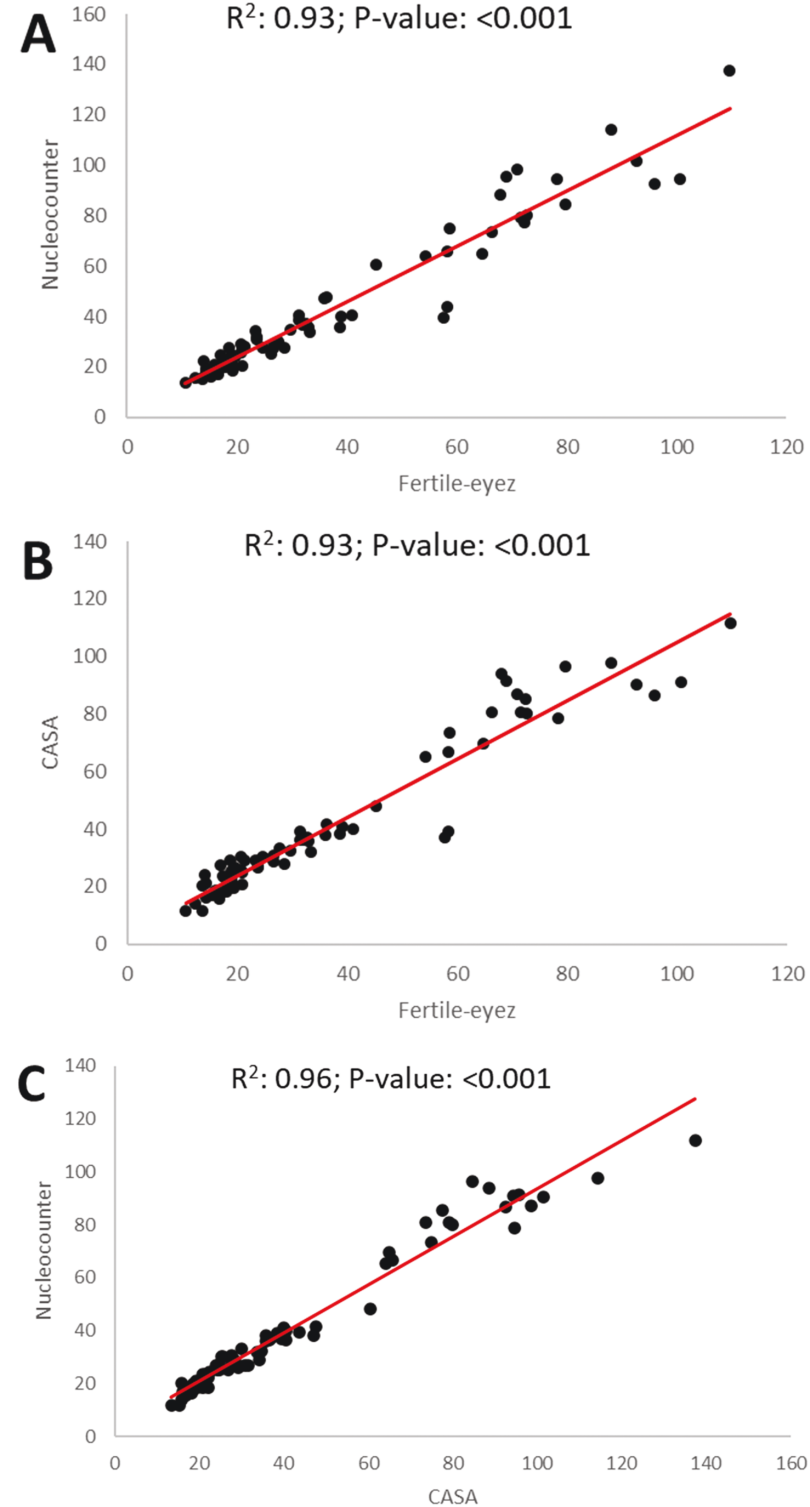
Linear regression between sperm concentration values measured with Fertile-Eyez, compared with Nucleocounter (A) and CASA (B), and between Nucleocounter and CASA (C).

Correlation analysis allowed evaluation of whether differences in concentration had similar variation for each of the three devices. Comparison between the three devices showed significant correlation (*P* < 0.05) when all dilutions were analyzed as well as when samples were separated by dilution factor (Table 2). All the devices were strongly correlated except for at the 1:8 dilution, which was moderately correlated, for the comparison between FE and NC, and between FE and CASA, and at the 1:16 dilution for FE and CASA. Representation of the concentration measured using NC and FE by dilution factor are shown in Figure 1. The lower *r* value at 1:8 dilution (*r* = 0.583) could be due to 2 or 3 data points that deviate from the regression line. Devices were also evaluated for accuracy using linear regression where a significant *P*-value (*P* < 0.05) indicates that the data is linear and the *R*^2^ value is interpreted as the percentage of the data variation explained by the linear model. Linear regression analysis showed that concentration measured with the three devices significantly (*P* < 0.001) fitted a linear model. In addition, R^2^ values >0.9 were found between FE and NC, and FE and CASA (Figure 2), which indicates that the three devices have similar variation in their measurements of concentration.

**Table 2.** Correlation among concentrations measured by NucleoCounter (NC), Fertile-Eyez (FE), and CASA in serial‐diluted swine semen samples

Method		Correlation (*r*)	*P*-value
All dilutions (*n* = 82)			
NC	FE	0.967	<0.0001
NC	CASA	0.982	<0.0001
FE	CASA	0.964	<0.0001
Dilution 1:4 (~82 × 10^6^cell/mL) (*n* =18)			
NC	FE	0.819	<0.0001
NC	CASA	0.880	<0.0001
FE	CASA	0.778	0.0001
Dilution 1:8 (~37 × 10^6^cell/mL) (n=18)			
NC	FE	0.583	0.011
NC	CASA	0.893	<0.0001
FE	CASA	0.615	0.007
Dilution 1:10 (~25 × 10^6^cell/mL) (*n* = 18)			
NC	FE	0.896	<0.0001
NC	CASA	0.839	<0.0001
FE	CASA	0.809	<0.0001
Dilution 1:16 (~18 × 10^6^cell/mL) (*n* = 18)			
NC	FE	0.705	0.001
NC	CASA	0.854	<0.0001
FE	CASA	0.641	0.004

**Figure 3.**
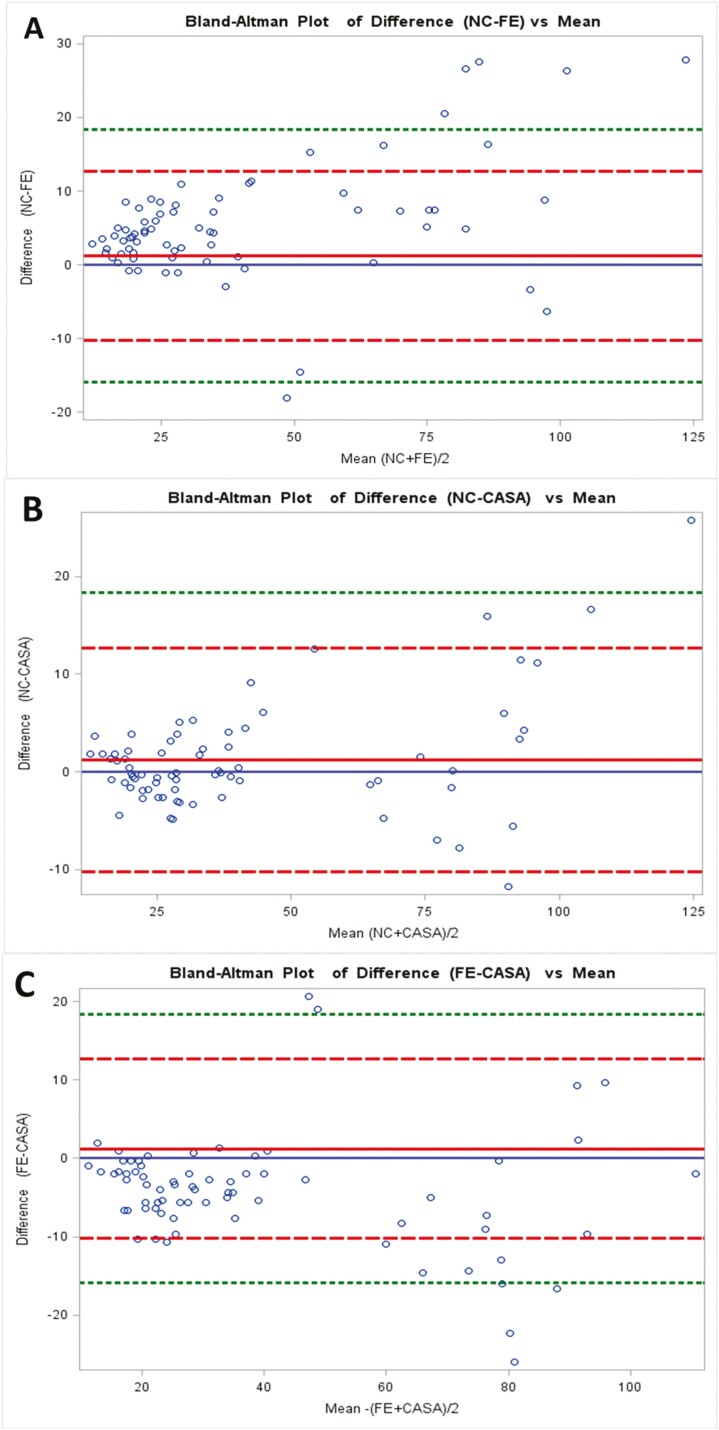
Bland-Altman plots of sperm concentration measured with Fertile-Eyez (FE), Nucleocounter (NC) and CASA in 1:4, 1:8, 1:10, and 1:16 serial-diluted swine semen. Analysis was performed for the match between (A) NC-FE; (B) NC-CASA; and (C) FE-CASA. The red solid line represents the average of the differences, blue solid line represents 0, the red dashed lines are the ±2× SD; and the green dashed lines represent ±3× SD. All values are given as 10^6^ cells/mL.

The Bland-Altman analysis is used to assess agreement between two evaluation methods (Bland and Altman, 1986). In the current study, Bland-Altman analysis showed similarity between NC and CASA at all dilutions (<10%; Figure 3). NC and FE, as well as CASA and FE, has the greatest similarity at the 1:8 dilution with slightly less similarity at the other dilutions (Table 3). Concentrations reported by NC and CASA were greater than those reported by FE at all dilutions with variation above 10% at all dilutions except 1:8.

**Table 3.** Bland-Altman analysis of the sperm concentration measured using NucleoCounter (NC), Fertile-Eyez (FE), and CASA in serial‐diluted swine semen samples

Method		Mean difference (%)
Dilution 1:4		
NC	FE	11.65(+14.2%)
NC	CASA	3.08(+3.7%)
FE	CASA	−8.57(−17.0%)
Dilution 1:8		
NC	FE	1.93(+5.2%)
NC	CASA	1.63(+4.5%)
FE	CASA	−0.30(−0.8%)
Dilution 1:10		
NC	FE	5.26(+21.9%)
NC	CASA	−0.24(−0.9%)
FE	CASA	−5.50(−22.8%)
Dilution 1:16		
NC	FE	2.87(+16.1%)
NC	CASA	0.43(+2.3%)
FE	CASA	−2.44(−13.9%)

Data represent the difference of the means between devices for the same dilution factors, and the percentage that the difference represents of the mean for sperm concentration.

The NC is the gold standard for accuracy and repeatability of measuring concentration in semen samples. The low CV for all dilutions for the NC supports this idea. The smartphone-based device (FE) showed similar results to the computer-based device (CASA) when used to measure sperm concentration, as demonstrated by similarity in repeatability and accuracy of measurements. FE results were accurate, as demonstrated by the high correlation with NC and CASA results. Finally, Bland-Altman analysis demonstrated that dilution 1:8 was most correlated among the three devices.

### Evaluation of FE as a Device to Measure Total Motility in Boar Semen

Testing of the accuracy of FE to measure total motility was assessed by comparing data measured in serial-diluted boar semen samples with results obtained by CASA. The average total motility, SD, and CV for both devices are presented in Table 4. The range in SD for the dilutions using CASA was 1.24–1.77 and for FE 0.89–1.18. The range in CV for CASA was 2.45–7.10 and for FE 3.46–6.03. Correlation analysis was significant when all dilutions were evaluated together (*P* < 0.001) and the correlation coefficient showed a moderated correlation (*r* = 0.558, Table 5). When each dilution was analyzed individually, only the 1:10 dilution was significant (*P* = 0.044) and moderately correlated (*r* = 0.479). The 1:8 dilution showed a tendency (*P* = 0.098) to correlate motility between FE and CASA with moderate correlation (*r* = 0.403). Lower coefficient of correlation values found for motility data may be a result of using samples with similar motility. The RSD calculated in the regression analysis showed that overall, the measurement of motility showed a 3.95% variation between FE and CASA. Semen samples ranged from a minimum motility value of 62.7% to a maximum value of 92.5% indicating that FE could measure data spanning values above and below the industry threshold of 70%. Previous authors that have found strong correlations (*r* > 0.70) when comparing devices for assessment of motility using frozen semen with more variation in motility for their analyses (10% to 60%, Dini et al., 2019; 0% to 80%, Kanakasabapathy et al., 2017). Therefore, the lower variation between samples most likely have influenced the correlation analysis, suggesting future studies should be performed with a wider range of motility that include sub-fertile boars with 50%–70% motility and less. Bland-Altman analysis showed that the mean difference between devices was equal or lower than 10% of the means, indicating high similarity in the measurement of motility between devices (Table 6).

**Table 4.** Mean total motility measured in serial-diluted boar samples using Fertile-Eyez and CASA

	CASA			Fertile-Eyez		
Dilution	Mean	SD	CV	Mean	SD	CV
1:4	84.00	1.24	2.45	82.57	0.93	3.46
1:8	80.66	1.77	6.08	81.52	0.89	6.08
1:10	81.35	1.62	7.10	80.00	1.18	6.03

**Table 5.** Correlation among total motility measured with Fertile-Eyez (FE) and CASA in serial‐diluted swine semen samples

Method		Correlation (*r*)	*P*-value
All dilutions			
Fertile-Eyez	CASA	0.558	< 0.001
Dilution 1:4			
Fertile-Eyez	CASA	0.043	0.866
Dilution 1:8			
Fertile-Eyez	CASA	0.403	0.098
Dilution 1:10			
Fertile-Eyez	CASA	0.479	0.044

**Table 6.** Bland-Altman analysis of the semen total motility measured using NucleoCounter, Fertile-Eyez, and CASA in serial‐diluted swine semen samples

Method		Mean difference (%)
Dilution 1:4		
FE	CASA	-3.37(-3.9%)
Dilution 1:8		
FE	CASA	-2.45(-3.0%)
Dilution 1:10		
FE	CASA	+3.34(+4.2%)

Data represent the difference of the means between devices for the same dilution actors, and the percentage that the difference represents of the mean of for sperm concentration.

### Evaluation of FE as a Device to Assess Sperm Morphology in Boar Semen

**Figure 4.**
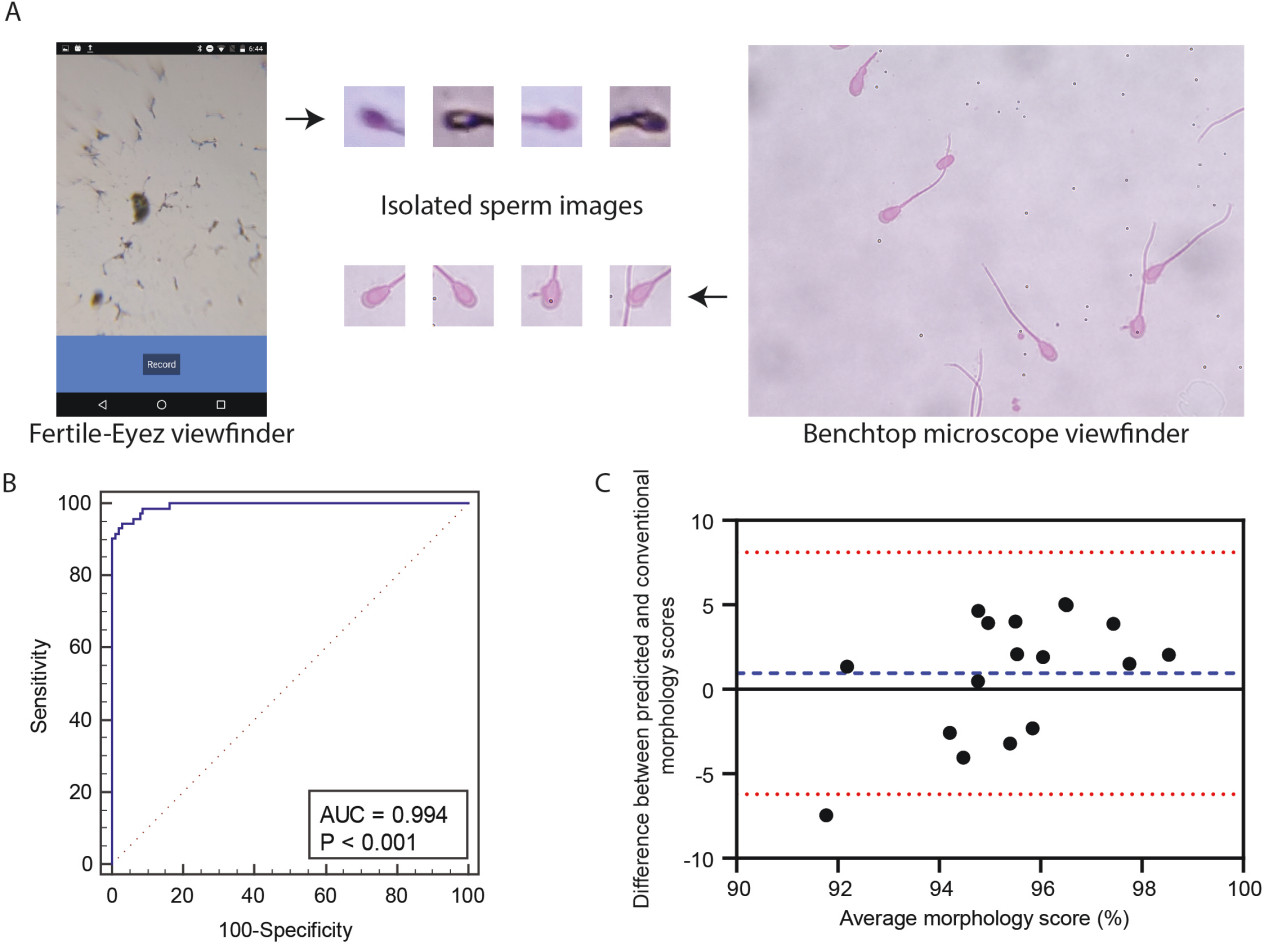
Evaluation of sperm morphology using Fertile-Eyez artificial intelligence. (A) Sperm images as imaged with a Fertile-Eyez device and a benchtop microscope. (B) Receiver operating characteristic curve when analyzing the artificial intelligence algorithm’s ability to differentiate sperm cells based on their morphological quality (normal vs. abnormal) (*n* = 270). (C) Bland-Altman plot comparing conventionally estimated morphology scores and the scores estimated by Fertile-Eyez (*n* = 17).

For morphological assessment, 17 samples were evaluated by the FE artificial intelligence algorithm, and its results were compared with manual counts obtained by a trained technician (Figure 4). Bland-Altman analysis showed that the difference between the two methods on average was 0.95, with its 95% limits of agreement ranging from -6.20% to 8.11%, indicating the similarity of measurement between the technician and the FE technology. The result indicated that the assessment of morphology by FE was like conventional assessments of morphology of expert human technicians.

## CONCLUSION

The repeatability and accuracy of using FE for evaluating concentration of boar semen samples were similar to NC and CASA, with the greatest accuracy at the 1:8 dilution. Despite being moderately correlated, the repeatability and accuracy of using FE for evaluating motility in boar semen samples were similar to CASA. Further studies, with a wider range of motilities, should be performed to further evaluate the precision of FE to assess sperm motility. FE artificial intelligence is also capable of performing automated morphology assessments of sperm cells similar to a trained expert technician. FE is a portable, smartphone-based device capable of assessing concentration, motility, and morphology of boar semen samples.

## References

[CIT0001] Amann, R. P., and D. Waberski. 2014. Computer-assisted sperm analysis (CASA): capabilities and potential developments. Theriogenology. 81:5–17.e11-13. doi:10.1016/j.theriogenology.2013.09.004. 24274405

[CIT0002] Bland, J. M., and D. G. Altman. 1986. Statistical methods for assessing agreement between two methods of clinical measurement. Lancet. 1:307–310. doi:10.1016/S0140-6736(86)90837-8. 2868172

[CIT0003] Bulkeley, E., C. Collins, A. Foutouhi, K. Gonzales, H. Power, and S. Meyers. 2021. Assessment of an iPad-based Sperm motility analyzer for determination of canine sperm motility. Transl. Anim. Sci. 5:txab066. doi:10.1093/tas/txab066. 34124591 PMC8191482

[CIT0004] Buss, T., J. Aurich, and C. Aurich. 2019. Evaluation of a portable device for assessment of motility in stallion semen. Reprod Domest Anim 54:514–519. doi:10.1111/rda.13390. 30592335 PMC7379573

[CIT0005] Camus, A., S. Camugli, C. Lévêque, E. Schmitt, and C. Staub. 2011. Is photometry an accurate and reliable method to assess boar semen concentration? Theriogenology 75:577–583. doi:10.1016/j.theriogenology.2010.09.025. 21074835

[CIT0006] Dini, P., L. Troch, I. Lemahieu, P. Deblende, and P. Daels. 2019. Validation of a portable device (iSperm^®^) for the assessment of stallion sperm motility and concentration. Reprod. Domest. Anim. 54:1113–1120. doi:10.1111/rda.13487. 31177582

[CIT0007] Flowers, W. L. 1997. Management of boars for efficient semen production. J. Reprod. Fertil. Suppl. 52:67–78. doi:9602720

[CIT0008] Hansen, C., T. Vermeiden, J. P. W. Vermeiden, C. Simmet, B. C. Day, and H. Feitsma. 2006. Comparison of FACSCount AF system, Improved Neubauer hemocytometer, Corning 254 photometer, SpermVision, UltiMate and NucleoCounter SP-100 for determination of sperm concentration of boar semen. Theriogenology 66:2188–2194. doi:10.1016/j.theriogenology.2006.05.020. 16920186

[CIT0009] Jasko, D. J. 1992. Evaluation of stallion semen. Vet. Clin. North Am. Equine Pract. 8:129–148. doi:10.1016/s0749-0739(17)30471-6. 1576546

[CIT0010] Kanakasabapathy, M. K., M. Sadasivam, A. Singh, C. Preston, P. Thirumalaraju, M. Venkataraman, C. L. Bormann, M. S. Draz, J. C. Petrozza, and H. Shafiee. 2017. An automated smartphone-based diagnostic assay for point-of-care semen analysis. Sci. Transl. Med. 9:eaai7863. doi:10.1126/scitranslmed.aai7863. 28330865 PMC5701517

[CIT0011] Kanakasabapathy, M. K., P. Thirumalaraju, H. Kandula, F. Doshi, A. D. Sivakumar, D. Kartik, R. Gupta, R. Pooniwala, J. A. Branda, A. M. Tsibris, et al. 2021. Adaptive adversarial neural networks for the analysis of lossy and domain-shifted datasets of medical images. Nat. Biomed. Eng. 5:571–585. doi:10.1038/s41551-021-00733-w. 34112997 PMC8943917

[CIT0012] Morrell, J. M., A. Johannisson, L. Juntilla, K. Rytty, L. Bäckgren, A. M. Dalin, and H. Rodriguez-Martinez. 2010. Stallion sperm viability, as measured by the nucleocounter sp-100, is affected by extender and enhanced by single layer centrifugation. Vet Med Int. 2010:659862. doi:10.4061/2010/659862. 20445788 PMC2860196

[CIT0013] Mortimer, S. T., G. van der Horst, and D. Mortimer. 2015. The future of computer-aided sperm analysis. Asian J. Androl. 17:545–553. doi:10.4103/1008-682X.154312. 25926614 PMC4492043

[CIT0014] Mukaka, M. M. 2012. Statistics corner: a guide to appropriate use of correlation coefficient in medical research. Malawi Med J. 24:69–71. doi:10.4314/MMJ.V24I3. 23638278 PMC3576830

[CIT0015] Ratner, B. 2009. The correlation coefficient: its values range between +1/−1, or do they? J Target. Meas. Anal. Mark. 17:139–142. doi:10.1057/jt.2009.5.

[CIT0016] Schober, P., C. Boer, and L. A. Schwarte. 2018. Correlation coefficients: appropriate use and interpretation. Anesth Analg. 126:1763–1768. doi:10.1213/ANE.0000000000002864. 29481436

[CIT0017] Thirumalaraju, P., C. Bormann, M. Kanakasabapathy, F. Doshi, I. Souter, I. Dimitriadis, and H. Shafiee. 2018. Automated sperm morpshology testing using artificial intelligence. Fertil. Steril. 110:e432. doi:10.1016/J.FERTNSTERT.2018.08.039.

[CIT0018] Thirumalaraju, P., M. K. Kanakasabapathy, C. L. Bormann, H. Kandula, S. K. Sai Pavan, D. Yarravarapu, and H. Shafiee. 2019. Human sperm morphology analysis using smartphone microscopy and deep learning. Fertil. Steril. 112:e41. doi:10.1016/j.fertnstert.2019.07.237.

[CIT0019] Zinaman, M. J., M. L. Uhler, E. Vertuno, S. G. Fisher, and E. D. Clegg. 1996. Evaluation of computer-assisted semen analysis (CASA) with IDENT stain to determine sperm concentration. J. Androl. 17:288–292. 8792219

